# Perceived neighborhood assets and life satisfaction among adolescents attending school in a rural insular community

**DOI:** 10.3389/fpsyg.2026.1992746

**Published:** 2026-10-14

**Authors:** Victor Manuel Pardo, Susana De La Ossa, Manuel Noreña, Daisy Silva

**Affiliations:** 1 Psychology Program, University of San Buenaventura, Cartagena, Colombia; 2 Department of Educational Studies, School of Arts and Humanities, University of Caldas, Manizales, Colombia

**Keywords:** adolescence, Colombia, life satisfaction, perceived neighborhood assets, rural island community, subjective wellbeing

## Abstract

Adolescents’ life satisfaction is embedded in social and physical environments that may provide support, safety, belonging, and opportunities for participation. This study examined whether perceived neighborhood assets were associated with life satisfaction after accounting for sociodemographic, relational, adaptive, and emotional characteristics. A cross-sectional study was conducted with 313 students aged 12–19 years (*M* = 14.26, SD = 1.80) attending a public school in a predominantly Afro-Colombian rural island community of Cartagena, Colombia. Participants completed measures of life satisfaction, perceived neighborhood assets, peer attachment, psychosocial adaptation, and perceived emotional intelligence. Scale scores were calculated using predefined item-completion criteria, and remaining missing scores were handled through multiple imputation by chained equations using 50 imputed datasets. Hierarchical linear regression was used to estimate the incremental contribution of perceived neighborhood assets. Age and sex explained 0.7% of the variance in life satisfaction. Adding peer attachment, psychosocial adaptation, Emotional Attention, Emotional Clarity, and Emotional Repair increased the explained variance to 3.7%, but this block did not significantly improve model fit, pooled *F*(5, 302.05) = 1.74, *p* = 0.125. Adding perceived neighborhood assets increased the explained variance to 6.4% (Δ*R*^2^ = 0.027) and significantly improved model fit, pooled *F*(1, 299.70) = 8.40, *p* = 0.004. A more favorable appraisal of neighborhood assets was associated with higher life satisfaction (*B* = 0.0456, SE = 0.0157, 95% CI [0.0146, 0.0765], β = 0.168, *p* = 0.004). Complete-case and observed-outcome sensitivity analyses produced comparable estimates. Perceived neighborhood assets showed a modest association with adolescents’ life satisfaction beyond the other characteristics considered. Longitudinal and multisite studies are needed to examine how structural conditions and adolescents’ experiences of their residential communities jointly relate to subjective wellbeing.

## 1 Introduction

Life satisfaction refers to the cognitive evaluation individuals make of their lives according to their own standards, expectations, and circumstances (Diener et al., 1985; Pavot and Diener, 2008). As the cognitive component of subjective wellbeing, it is conceptually distinct from transient affective states, psychological symptoms, and evaluations restricted to particular life domains. During adolescence, judgments about life satisfaction develop alongside changes in autonomy, identity, interpersonal relationships, and participation in broader social settings. Their assessment provides information about how young people evaluate their lives from their own perspective, beyond inferences based solely on the absence of psychological or behavioral difficulties.

Ecological and positive youth development approaches locate these evaluations within the environments in which adolescents live. Young people interact daily with families, peers, schools, residential communities, and local institutions, and the resources available across these settings may support or constrain developmental opportunities (Bronfenbrenner, 1979; Benson et al., 2011). From a developmental-assets perspective, internal assets include personal competencies, whereas external assets comprise the support, relationships, opportunities, and conditions provided by adolescents’ social environments. Residential communities form part of this external resource system because they shape opportunities for interaction, participation, mobility, recreation, and access to supportive adults and institutions.

Perceived neighborhood assets refer to adolescents’ evaluations of community characteristics that may support their development and adjustment. The construct examined in this study encompasses youth support and empowerment, neighborhood attachment, perceived safety, informal social control, and activities for young people (Oliva et al., 2012). These components reflect whether adolescents feel valued and able to participate, experience belonging and safety, perceive adults as willing to respond to community problems, and have access to constructive recreational and social opportunities. A global appraisal across these components summarizes how supportive adolescents perceive their residential environment to be.

Adolescents’ reports of neighborhood conditions provide information that is related to, but not interchangeable with, structural indicators. Objective indicators describe socioeconomic composition, infrastructure, access to public services, or officially recorded violence, whereas perceived neighborhood assets reflect how young people experience and interpret their social and physical surroundings. Objective and subjective measures may therefore show different associations with individual outcomes (Weden et al., 2008). Structural neighborhood conditions may also be related to adolescent behavioral and emotional outcomes through perceptions of social resources and neighborhood disorder (Yoon, 2021).

Young people living in the same territory may differ in their sense of belonging, perceived safety, access to activities, and opportunities for participation. These perceptions can influence whether local spaces are used, avoided, or experienced as supportive. Steenwegen et al. (2026), in a systematic review of 32 studies, identified social cohesion, perceived safety, peer relationships, adult support, accessible public spaces, and community institutions as potential resources for children’s wellbeing. Associations varied according to age, socioeconomic background, cultural setting, and resource accessibility. Although the review focused primarily on children aged 6–12 years, it covered developmental periods immediately preceding and partly overlapping with early adolescence and emphasized the importance of young people’s perspectives and agency.

Studies focused on adolescents have also connected neighborhood experiences with life satisfaction. Among 2,400 Spanish adolescents, neighborhood assets—including youth empowerment, neighborhood attachment, perceived safety, and social control—were associated with adjustment and life satisfaction (Delgado et al., 2012). Research with Chilean adolescents found that sense of community was associated with global life satisfaction (Guzmán et al., 2019) and mediated the relationship between perceived community support and life satisfaction (Alfaro et al., 2017). Perceived safety at home and in the community has also been associated with adolescent life satisfaction directly and indirectly through neighborhood satisfaction (Varela et al., 2020). Multilevel evidence from England indicates that only a small proportion of variation in adolescent life satisfaction occurs between neighborhoods and that associations with specific neighborhood characteristics depend on the indicators and model specifications examined (Marquez et al., 2024). Neighborhood contributions to adolescent wellbeing may therefore be modest, multidimensional, and sensitive to how the residential environment is operationalized.

Several features of everyday experience may connect neighborhood appraisal with life satisfaction. Perceived safety can facilitate mobility and participation in local spaces; attachment can provide continuity and belonging; recreational and participatory opportunities can expand social involvement and constructive leisure; and informal support may provide resources extending beyond adolescents’ households. The relevance of these experiences is likely to vary across social, cultural, and territorial settings, limiting direct generalization from urban European or North American populations to communities with different histories, geographies, and access to institutional resources.

Evidence from Latin America remains comparatively limited. Studies with Chilean adolescents have examined community support, sense of community, safety, neighborhood satisfaction, and life satisfaction (Alfaro et al., 2017; Guzmán et al., 2019; Varela et al., 2020). Qualitative research in Bogotá has described how young people construct relationships with the places they inhabit, illustrating the role of the residential environment in their everyday experiences and subjectivities (Joya Valbuena, 2021). This literature supports attention to adolescents’ subjective relationships with place while showing that those experiences remain embedded in locally specific social and spatial conditions.

Colombian research on adolescent wellbeing has more frequently examined personal, emotional, family, and behavioral resources. In a sample of 930 adolescents attending schools in Bogotá and Medellín, emotion regulation, proactive coping, and prosocial behavior were associated with wellbeing and life satisfaction (Ripoll-Núñez et al., 2020). Research conducted in Cartagena has examined personal and socio-emotional competencies in relation to psychosocial adaptation among secondary-school students from socially disadvantaged settings (Pardo et al., 2025). These studies provide evidence about positive functioning among Colombian adolescents, but quantitative research examining perceived neighborhood resources in relation to global life satisfaction remains scarce, particularly in rural island communities.

The association between perceived neighborhood assets and life satisfaction should also be examined alongside other resources that may shape adolescents’ evaluations of their lives. Peer attachment reflects trust, communication, and alienation within relationships that acquire increasing developmental salience during adolescence. Attachment to parents and peers has been associated with life satisfaction during middle childhood and early adolescence, although the magnitude of these relationships varies across grade levels and life domains (Nickerson and Nagle, 2004). Psychosocial adaptation provides a broader indicator of functioning in study activities, family and social relationships, leisure, participation, and engagement with the surrounding environment. Accounting for these characteristics helps distinguish appraisal of the broader residential community from adolescents’ immediate peer relationships and general social functioning.

Perceived emotional intelligence constitutes another set of personal resources potentially associated with life satisfaction. Within the Trait Meta-Mood framework, it comprises attention to emotional experiences, clarity in understanding emotional states, and perceived ability to regulate negative moods (Salovey et al., 1995; Fernández-Berrocal et al., 2004). A systematic review and meta-analysis of 41 studies found positive associations between emotional intelligence and both cognitive and affective components of subjective wellbeing in adolescents (Llamas-Díaz et al., 2022). Longitudinal evidence has also linked perceived emotional intelligence with later life satisfaction through positive and negative affect (Sánchez-Álvarez et al., 2015). The dimensions may not relate to wellbeing uniformly: clarity and repair generally show favorable associations, whereas emotional attention may accompany rumination or distress when it is not supported by adequate understanding and regulation.

Considering peer attachment, psychosocial adaptation, and perceived emotional intelligence provides a more stringent assessment of the neighborhood association. An observed relationship between neighborhood appraisal and life satisfaction might otherwise reflect adolescents’ interpersonal adjustment, broader functioning, or emotional self-perceptions. Statistical adjustment cannot determine temporal or causal direction in cross-sectional data, but it can indicate whether perceived neighborhood assets retain a concurrent association with life satisfaction after these characteristics are considered.

This question is particularly relevant in rural island settings, where geographic isolation and unequal access to transportation, sanitation, public services, education, healthcare, and recreational opportunities may shape adolescents’ daily environments. The present study was conducted in a predominantly Afro-Colombian rural community on Tierra Bomba Island, Cartagena. Territorial research has documented socioeconomic and infrastructural disparities between Cartagena’s island communities and its urban area (Jiménez-Castilla et al., 2018; Cartagena Cómo Vamos, 2021). These aggregate conditions characterize the broader setting but do not establish the socioeconomic circumstances or ethnic self-identification of individual participants. Assessing perceived neighborhood assets complements area-level indicators by examining how adolescents evaluate the support, safety, belonging, supervision, and opportunities available in their residential environments.

The present study examined whether perceived neighborhood assets accounted for additional variance in life satisfaction beyond age, sex, peer attachment, psychosocial adaptation, and perceived emotional intelligence among adolescents attending a public school in a rural island community of Cartagena, Colombia. We expected a more favorable global appraisal of neighborhood assets to be positively associated with life satisfaction after accounting for these concurrent demographic, relational, adaptive, and emotional characteristics.

## 2 Materials and methods

### 2.1 Study design

This study employed a quantitative, cross-sectional, correlational design to examine the incremental contribution of perceived neighborhood assets to adolescents’ life satisfaction.

### 2.2 Setting and participants

The study was conducted at a public school in a predominantly Afro-Colombian rural community on Tierra Bomba Island, Cartagena, Colombia. Access to mainland Cartagena is primarily maritime, and the local economy has traditionally included artisanal fishing and informal economic activities. Territorial studies have documented disadvantages affecting the island’s communities in sanitation, public services, healthcare, education, transportation, and exposure to environmental risks (Jiménez-Castilla et al., 2018; Cartagena Cómo Vamos, 2021; Del Cairo Hurtado et al., 2023). This contextual information was used solely to describe the study setting and not to assign socioeconomic status or ethnic identity to individual participants.

Participants were recruited through non-probability convenience sampling under an interinstitutional agreement with the school. After the research team presented the study to the school principal, the school administration informed teachers, students, parents, and legal guardians about the project and facilitated the distribution and collection of consent forms. Students from nine intact classes in grades 7–11 were invited to participate: two classes from each of grades 7–10 and one class from grade 11. Both morning and afternoon school schedules were represented, although classroom and schedule identifiers were not retained in the analytical dataset.

The sample comprised 313 students aged 12–19 years (*M* = 14.26, SD = 1.80), including 163 girls (52.1%) and 150 boys (47.9%). Participants were enrolled in seventh (*n* = 72, 23.0%), eighth (*n* = 74, 23.6%), ninth (*n* = 85, 27.2%), tenth (*n* = 53, 16.9%), or eleventh grade (*n* = 29, 9.3%). Age, sex, and grade data were available for all participants. Sex was self-reported using the response categories male and female; gender identity was not assessed.

Most students were understood to reside in the local community, although a small, unquantified number commuted daily from a neighboring community on the island. Place of residence, length of residence, time spent in the residential community, and mobility outside it were not recorded. The participants should therefore be considered a school-based sample rather than an exclusively residential sample.

Students were eligible if they were enrolled in one of the participating classes and fulfilled the applicable consent requirements. Written parental or legal guardian consent and adolescent assent were required for participants younger than 18 years; participants aged 18 years or older provided written informed consent on their own behalf. All students who were present during data collection and had completed the applicable consent process agreed to participate. The numbers of absent students and students who did not return consent forms were not recorded, precluding calculation of a participation rate relative to the total eligible school population. Although this study and Pardo et al. (2025) arose from the same broader research project, the present sample was collected at a different institution and during a different period; therefore, no participants overlapped between the two studies.

### 2.3 Measures

#### 2.3.1 Satisfaction with life

Life satisfaction was assessed using the Satisfaction With Life Scale (SWLS; Diener et al., 1985). The scale comprises five items that assess a person’s global cognitive evaluation of life, including satisfaction with current life conditions and perceived attainment of important goals. Although the original SWLS uses seven response categories, the form administered in this study used five categories ranging from 1 (*totally disagree*) to 5 (*totally agree*), consistent with five-category Spanish-language applications of the scale (Atienza et al., 2000). This response format was administered uniformly to all participants. Item scores were summed to produce a total score ranging from 5 to 25, with higher scores indicating greater life satisfaction. In the present sample, the SWLS showed acceptable internal consistency (Cronbach’s α = 0.752; polychoric McDonald’s ω_t_ = 0.804). These coefficients were estimated from the 295 participants who completed all five items.

#### 2.3.2 Perceived neighborhood assets

Perceived neighborhood assets were assessed using the Scale for the Assessment of Developmental Assets in the Neighborhood developed by Oliva et al. (2012). The instrument contains 22 items rated from 1 (completely false) to 7 (completely true) and assesses five components: youth support and empowerment, neighborhood attachment, perceived safety, informal social control, and activities for young people.

Before completing the instrument, participants were instructed to interpret “neighborhood” as the residential community in which they lived. The four negatively oriented safety items were reverse-scored. The primary analysis used the global score obtained by summing the 22 items, with possible values ranging from 22 to 154 and higher scores representing a more favorable overall appraisal of neighborhood assets. The global score showed adequate internal consistency (α = 0.820; polychoric ω_t_ = 0.842), based on 276 participants with complete item-level data.

The five component scores were examined in a post hoc exploratory analysis. Reliability varied across components: youth support and empowerment (α = 0.787, ω_t_ = 0.809), neighborhood attachment (α = 0.809, ω_t_ = 0.841), perceived safety (α = 0.702, ω_t_ = 0.784), informal social control (α = 0.582, ω_t_ = 0.672), and activities for young people (α = 0.483, ω_t_ = 0.523). Results involving the latter two components were interpreted cautiously because of their limited internal consistency.

#### 2.3.3 Peer attachment

Peer attachment was assessed using the Spanish adaptation of the peer scale from the Inventory of Parent and Peer Attachment (Armsden and Greenberg, 1987; Sánchez-Queija and Oliva, 2003). The instrument contains 21 items rated from 1 (completely disagree) to 7 (completely agree) and comprises Trust, Communication, and Alienation subscales.

Following the scoring instructions, the global score was calculated by adding Trust and Communication and subtracting Alienation. Higher scores represented more favorable peer attachment. For reliability estimation, the Alienation items were reverse-oriented so that all items followed the same direction. The global score showed adequate internal consistency (α = 0.833; polychoric ω_t_ = 0.884), based on 268 participants with complete responses to all 21 items.

#### 2.3.4 Psychosocial adaptation

Psychosocial adaptation was assessed using the Social Adaptation Self-evaluation Scale (SASS; Bosc et al., 1997). The instrument assesses perceived functioning in study or work, household activities, leisure, family and social relationships, intellectual interests, community participation, and management of everyday social demands. The original instrument begins with two alternative items concerning paid work or household activities. Because the participants were school students, the household-activities item was administered, and each participant completed 20 scored items.

Responses were rated from 0 to 3. The three negatively oriented items concerning difficulty expressing opinions, perceived rejection or exclusion, and difficulty managing financial resources were reverse-scored before calculating the total. Scores could range from 0 to 60, with higher values indicating greater psychosocial adaptation. The correctly scored total showed acceptable internal consistency (α = 0.783; polychoric ω_t_ = 0.843). Complete item-level data were available for all 313 participants.

#### 2.3.5 Perceived emotional intelligence

Perceived emotional intelligence was assessed using the Trait Meta-Mood Scale-24 (TMMS-24; Fernández-Berrocal et al., 2004), an abbreviated Spanish version of the original Trait Meta-Mood Scale (Salovey et al., 1995). The instrument contains 24 items rated from 1 (completely disagree) to 5 (completely agree).

The TMMS-24 produces three eight-item scores. Emotional Attention assesses the extent to which individuals attend to their emotional states; Emotional Clarity assesses their perceived understanding of those states; and Emotional Repair assesses their perceived ability to regulate negative moods and maintain or restore more favorable emotional states. Each dimension ranges from 8 to 40. The dimensions were analyzed separately because they represent distinguishable emotional self-perceptions.

Internal consistency was adequate for Emotional Attention (α = 0.818; polychoric ω_t_ = 0.858), Emotional Clarity (α = 0.837; polychoric ω_t_ = 0.864), and Emotional Repair (α = 0.826; polychoric ω_t_ = 0.859). Reliability estimates were based on participants with complete responses within each dimension: 307 for Attention, 304 for Clarity, and 235 for Repair.

### 2.4 Procedure

The questionnaires were administered in paper-and-pencil format during two classroom sessions of approximately 45 min each. Administration was conducted collectively, although students completed the questionnaires individually. Each classroom session was attended by the class teacher, one member of the research team, and at least two trained psychology students serving as research assistants. The teacher facilitated the scheduled activity but did not administer the questionnaires. The research assistants had received prior training in the administration protocol, and several had previous experience supporting school-based data collection.

At the beginning of each session, the researcher explained the purpose of the study, the voluntary nature of participation, the confidentiality of responses, and participants’ right to discontinue without academic or personal consequences. Standardized instructions were provided. The researcher and research assistants remained available to clarify response procedures and unfamiliar wording without suggesting answers or interpreting the substantive meaning of the items for participants.

The available Spanish-language versions of the instruments were administered without further linguistic modification. However, no cognitive interviews, formal cross-cultural adaptation procedure, or measurement-invariance analysis was conducted specifically for the predominantly Afro-Colombian rural island population included in this study. In the printed questionnaire, the final TMMS-24 item belonged to the Emotional Repair dimension and appeared separately from the preceding items because of the page layout.

### 2.5 Ethical considerations

The study followed the ethical principles for research involving human participants established in the Declaration of Helsinki and the Colombian regulations applicable to health and psychological research. Participation was voluntary, and questionnaire responses were processed without personal identifiers in the analytical dataset. Written informed consent and adolescent assent were obtained before participation. The studies involving humans were approved by Bioethics Committee of the Universidad de San Buenaventura Cartagena.

### 2.6 Data preparation and missing-data treatment

The dataset was screened for invalid values, out-of-range responses, missing items, and consistency with the scoring instructions for each measure. No response-time data were available, and no additional screening based on invariant or repetitive response patterns was conducted. The four negatively oriented neighborhood-safety items and the three negatively oriented SASS items were reverse-scored before calculating their respective totals. Peer attachment was calculated by adding the Trust and Communication subscale scores and subtracting the Alienation subscale score. SWLS responses were oriented so that higher scores represented greater life satisfaction.

Scale scores were calculated when participants met the item-completion criterion established for the corresponding measure. Peer attachment required responses to at least 80% of the items in each subscale: seven of eight Trust items, six of seven Communication items, and five of six Alienation items. The neighborhood-assets score required at least 20 of the 22 items, each TMMS-24 dimension required at least seven of eight items, and the SWLS required at least four of five items. The SASS contained no missing item responses.

When the completion criterion was satisfied but one or more permissible items were missing, the mean of the available items was multiplied by the total number of items in the corresponding scale or subscale. Scores were left missing when the criterion was not met.

Scores remaining missing after prorating were handled using multiple imputation by chained equations. The imputation model included age, sex, peer attachment, perceived neighborhood assets, psychosocial adaptation, Emotional Attention, Emotional Clarity, Emotional Repair, and life satisfaction. Fifty imputed datasets were generated over 20 iterations. Predictive mean matching with five donors was used for variables containing missing values. This method was selected to preserve plausible observed values and reduce the possibility of imputations falling outside the empirical ranges of the measures. Multiple imputation assumed that the remaining values were missing at random conditional on the variables included in the imputation model. Convergence and plausibility were evaluated by examining trace plots, comparing the distributions of observed and imputed values, inspecting the ranges of the imputed scores, and reviewing logged events generated by the algorithm.

### 2.7 Reliability analysis

Internal consistency was evaluated using Cronbach’s alpha (α) and McDonald’s total omega (ω_t_). Alpha was calculated from the observed item scores, whereas omega was estimated from polychoric correlation matrices to account for the ordinal response formats. Reliability analyses were based on participants with complete responses to all items in the corresponding scale or component; consequently, the effective sample size varied across measures. All items were scored according to the applicable scoring instructions before reliability estimation. For multidimensional global scores, reliability was interpreted as the consistency of the composite score rather than as evidence of unidimensionality.

### 2.8 Statistical analysis

Descriptive statistics were calculated for the sociodemographic characteristics and principal study variables. Continuous variables were summarized using means, standard deviations, medians, interquartile ranges, and observed ranges. Categorical variables were described using frequencies and percentages.

Bivariate associations among the principal variables were examined using Spearman’s rank correlations because the scale scores were bounded and some showed departures from normality. The resulting *p*-values were adjusted across the 21 pairwise comparisons using the Benjamini–Hochberg false discovery rate procedure.

The primary analysis consisted of hierarchical linear regression across the 50 imputed datasets, with life satisfaction as the dependent variable. Model 1 included age and sex. Model 2 added peer attachment, psychosocial adaptation, Emotional Attention, Emotional Clarity, and Emotional Repair. Model 3 added the global perceived neighborhood-assets score. This ordering was specified to examine whether perceived neighborhood assets accounted for additional variance in life satisfaction after demographic characteristics and the included relational, adaptive, and emotional variables were considered.

Regression coefficients and standard errors were combined across imputations using Rubin’s rules. The incremental contribution of each block was evaluated using pooled D_1_ tests for nested multiply imputed models. Pooled R^2^ and adjusted R^2^ estimates were obtained for each model using the *pool.r.squared* procedure in *mice*. Confidence intervals for pooled R^2^ were derived by pooling the Fisher-transformed multiple-correlation estimates across imputations and transforming the resulting limits back to the R^2^ metric. Differences between pooled R^2^ point estimates were reported as descriptive estimates of ΔR^2^; inferential conclusions about the contribution of each block were based on the pooled D_1_ tests. Unstandardized coefficients, standardized coefficients, pooled standard errors, 95% confidence intervals, and two-sided *p*-values were reported. Statistical significance was defined as *p* < 0.05.

A sensitivity analysis estimated the smallest effect detectable with 80% power at a two-sided significance level of 0.05, given the sample size and number of predictors in the final model. An additional imputation-based analysis restricted the regression models to the 301 participants whose SWLS scores were originally observed rather than imputed. This analysis examined whether imputing the dependent variable materially affected the estimated association between perceived neighborhood assets and life satisfaction.

A complete-case analysis was also conducted using the 289 participants with complete information on all variables in the final model. Participants included and excluded from this analysis were compared on age using Welch’s *t*-test and on sex using Fisher’s exact test. Grade distributions were compared using Fisher’s exact test with Monte Carlo simulation because some expected frequencies were small. The complete-case final model was estimated using HC3 heteroskedasticity-consistent standard errors.

Linearity, residual distributions, homoscedasticity, multicollinearity, and influential observations were examined in the complete-case model. Homoscedasticity was evaluated using the Breusch–Pagan test, and multicollinearity was assessed using variance inflation factors. Cook’s distances were inspected using 4/*n* as a screening threshold. Observations exceeding this threshold were examined but were not removed solely because they crossed the cutoff. An added-variable plot based on residualized variables was used to examine visually the functional form of the association between perceived neighborhood assets and life satisfaction and the distribution of potentially influential observations.

In a post hoc exploratory analysis, the global neighborhood-assets score was replaced by its five component scores: youth support and empowerment, neighborhood attachment, perceived safety, informal social control, and activities for young people. The joint contribution of this five-component block was evaluated using a pooled D_1_ test. Component-specific coefficients and bivariate correlations were also examined. Benjamini–Hochberg corrections were applied separately to the five component-specific regression coefficients and the five component–SWLS correlations. Because this analysis was not prespecified and some component scores showed limited reliability, its results were interpreted as hypothesis-generating.

Participants were drawn from nine intact classes, but classroom and school-schedule identifiers were not retained in the analytical dataset. Consequently, within-class dependence could not be estimated and cluster-adjusted standard errors could not be calculated.

Analyses were conducted in R version 4.4.3 (R Core Team, 2025). Multiple imputation was performed using *mice* version 3.19.0 (van Buuren and Groothuis-Oudshoorn, 2011). Regression diagnostics were conducted using *car* (Fox and Weisberg, 2019) and *lmtest* (Zeileis and Hothorn, 2002). HC3 standard errors were estimated using *sandwich* (Zeileis, 2004; Zeileis et al., 2020), and internal consistency was evaluated using *psych* (Revelle, 2026).

## 3 Results

### 3.1 Scale-score availability and multiple imputations

After application of the item-completion and prorating rules, scale-score missingness ranged from 0% to 3.83%. Scores remained unavailable for peer attachment in three participants (0.96%), perceived neighborhood assets in one (0.32%), Emotional Attention in one (0.32%), Emotional Repair in eight (2.56%), and life satisfaction in 12 (3.83%). Psychosocial adaptation and Emotional Clarity scores were available for all participants. Complete data for all variables included in the primary regression models were available for 289 participants (92.33%).

The remaining missing scores were handled using 50 multiply imputed datasets generated over 20 iterations. The imputation algorithm produced no logged events. Visual inspection of the trace plots showed no persistent trends or systematic separation among chains. For Emotional Repair and life satisfaction, the variables with the largest numbers of missing scores, the observed and imputed distributions showed substantial overlap. Distributional comparisons for peer attachment, perceived neighborhood assets, and Emotional Attention were less informative because only three, one, and one scores, respectively, required imputation. All imputed scores fell within the corresponding observed ranges (Supplementary Figure 1).

### 3.2 Descriptive statistics and internal consistency

Descriptive statistics were calculated from the available observed scale scores before multiple imputation. Mean life satisfaction was 17.09 (SD = 4.79) on the 5–25 scale. The mean perceived neighborhood-assets score was 94.29 (SD = 17.71), and the mean peer-attachment score was 59.32 (SD = 17.46). Mean psychosocial adaptation was 34.48 (SD = 9.13), with a median of 35 and an observed range of 8–55. Descriptive statistics and internal-consistency coefficients for all study measures are presented in Table 1.

**TABLE 1:** Descriptive statistics and internal consistency of the principal study variables.

Variable	Score *n*	*M*	*SD*	Median	Observed range	α	ω_t_	Reliability *n*
Peer attachment	310	59.32	17.46	61	2–98	0.833	0.884	268
Perceived neighborhood assets	312	94.29	17.71	96	40–133	0.820	0.842	276
Psychosocial adaptation	313	34.48	9.13	35	8–55	0.783	0.843	313
Emotional Attention	312	26.82	6.96	28	8–40	0.818	0.858	307
Emotional Clarity	313	27.37	7.18	28	11–40	0.837	0.864	304
Emotional Repair	305	28.67	6.89	29	13.71–40	0.826	0.859	235
Life satisfaction	301	17.09	4.79	18	5–25	0.752	0.804	295

Descriptive statistics were calculated from the available observed scale scores after applying the prespecified item-completion and prorating rules and before multiple imputation. Score *n* indicates the number of participants with an available scale score. Reliability *n* indicates the number of participants with complete responses to all items included in the corresponding reliability analysis. Cronbach’s α was estimated from the observed item scores, and McDonald’s total omega (ω_t_) was estimated from polychoric correlation matrices.

### 3.3 Bivariate associations

Spearman correlations among the principal study variables are presented in Table 2. Perceived neighborhood assets showed a small positive correlation with life satisfaction, ρ = 0.210, *n* = 301, unadjusted *p* = 0.0002. This association remained statistically significant after Benjamini–Hochberg correction, adjusted *p* = 0.0013.

**TABLE 2:** Spearman correlations among the principal study variables.

Variable	1	2	3	4	5	6	7
1. Peer attachment	–	–	–	–	–	–	–
2. Perceived neighborhood assets	0.095	–	–	–	–	–	–
3. Psychosocial adaptation	0.026	0.040	–	–	–	–	–
4. Emotional Attention	−0.035	−0.010	−0.017	–	–	–	–
5. Emotional Clarity	−0.036	0.020	−0.100	0.651***	–	–	–
6. Emotional Repair	0.010	−0.037	−0.077	0.548***	0.714***	–	–
7. Life satisfaction	0.112	0.210**	0.029	0.112	0.094	0.089	–

**Adjusted *p* < 0.01.

***Adjusted *p* < 0.001. Values are Spearman’s rank-order correlation coefficients (ρ), calculated using pairwise-complete observations. Sample sizes varied across correlations because of missing scale scores (*n* = 293–313). *P*-values were adjusted across the 21 correlations using the Benjamini–Hochberg procedure.

Peer attachment, ρ = 0.112, unadjusted *p* = 0.055, and Emotional Attention, ρ = 0.112, unadjusted *p* = 0.053, showed small positive correlations with life satisfaction, but neither association remained statistically significant after correction for multiple comparisons, adjusted *p* = 0.191 for both. Life satisfaction was not significantly correlated with psychosocial adaptation, ρ = 0.029, Emotional Clarity, ρ = 0.094, or Emotional Repair, ρ = 0.089.

The three emotional dimensions were moderately to strongly intercorrelated. Emotional Attention correlated with Emotional Clarity, ρ = 0.651, and Emotional Repair, ρ = 0.548, whereas Emotional Clarity correlated with Emotional Repair, ρ = 0.714. All three correlations remained statistically significant after Benjamini–Hochberg correction, adjusted *p* < 0.001. Correlations among the remaining predictors were small and did not remain statistically significant after correction.

### 3.4 Hierarchical regression analysis

Hierarchical linear regression models were estimated across the 50 imputed datasets, and the resulting coefficients and standard errors were combined using Rubin’s rules. Age and sex were entered in Model 1. Peer attachment, psychosocial adaptation, Emotional Attention, Emotional Clarity, and Emotional Repair were added in Model 2. Perceived neighborhood assets were added in Model 3.

The sociodemographic model accounted for 0.72% of the variance in life satisfaction, R^2^ = 0.0072, adjusted R^2^ = 0.0003. Neither age nor sex was independently associated with life satisfaction. Adding the relational, adaptive, and emotional variables increased the explained variance to 3.67%, R^2^ = 0.0367, adjusted R^2^ = 0.0144, representing a descriptive ΔR^2^ of 0.0295. The joint contribution of this block was not statistically significant, pooled D_1_ *F*(5, 302.05) = 1.74, *p* = 0.125.

Adding perceived neighborhood assets increased the pooled explained variance from 3.67% to 6.36%, R^2^ = 0.0636, 95% CI [0.0206, 0.1258], adjusted R^2^ = 0.0388. The descriptive increment was ΔR^2^ = 0.0269, and the pooled comparison indicated that the addition of perceived neighborhood assets significantly improved the model, D_1_ *F*(1, 299.70) = 8.40, *p* = 0.004.

In the final model, a more favorable appraisal of neighborhood assets was independently associated with greater life satisfaction, *B* = 0.0456, SE = 0.0157, β = 0.168, 95% CI for *B* [0.0146, 0.0765], 95% CI for β [0.054, 0.283], *p* = 0.004. With the other predictors held constant, a 10-point difference in the neighborhood-assets score corresponded to an estimated 0.46-point difference in life satisfaction. A one-standard-deviation difference in perceived neighborhood assets corresponded to an estimated difference of approximately 0.80 points on the life-satisfaction scale.

Age, sex, peer attachment, psychosocial adaptation, Emotional Attention, Emotional Clarity, and Emotional Repair were not independently associated with life satisfaction in the final model. Their standardized coefficients ranged from −0.068 for age to 0.090 for peer attachment, and all corresponding confidence intervals included zero. The complete final-model coefficients are presented in Table 3.

**TABLE 3:** Final hierarchical regression model predicting life satisfaction after multiple imputation.

Predictor	*B*	SE	95% CI for *B*	β	95% CI for β	*P*
Intercept	10.273	3.663	[3.064, 17.482]	–	–	0.005
Age	−0.180	0.164	[−0.503, 0.143]	−0.068	[−0.189, 0.054]	0.273
Sex: girl	−0.467	0.544	[−1.538, 0.604]	−0.049	[−0.161, .063]	0.391
Peer attachment	0.025	0.016	[−0.007, 0.057]	0.090	[−0.026, 0.206]	0.127
Psychosocial adaptation	−0.008	0.031	[−0.070, 0.054]	−0.015	[−0.133, 0.102]	0.800
Emotional Attention	0.057	0.051	[−0.044, 0.157]	0.082	[−0.064, 0.228]	0.269
Emotional Clarity	−0.005	0.061	[−0.126, 0.116]	−0.008	[−0.189, 0.173]	0.931
Emotional Repair	0.054	0.059	[−0.062, 0.171]	0.078	[−0.089, 0.245]	0.360
Perceived neighborhood assets	0.0456	0.0157	[0.0146, 0.0765]	0.168	[0.054, 0.283]	0.004

Estimates were pooled across 50 imputed datasets using Rubin’s rules. *B*, unstandardized regression coefficient; SE, pooled standard error; β, standardized regression coefficient; CI, confidence interval. Male was the reference category for sex. All continuous predictors were entered in their original metric when estimating *B* and standardized before estimating β.

Model-level explained variance and pooled comparisons are summarized in Table 4. Across the hierarchical sequence, R^2^ increased from 0.0072 in the sociodemographic model to 0.0367 after addition of the relational, adaptive, and emotional variables and to 0.0636 after perceived neighborhood assets were entered. Only the contribution of the final block was statistically significant.

**TABLE 4:** Explained variance and pooled comparisons of the hierarchical regression models.

Model	Block added	R^2^	95% CI for R^2^	Adj_R^2^	Δ R^2^	Pooled D_1_ test, *F* (df1, df2)	*P*
1. Sociodemographic	Age and sex	0.0072	[0.0008, 0.0384]	0.0003	–	–	–
2. Relational, adaptive, and emotional	PA, PsyAd, EA, EC, ER	0.0367	[0.0064, 0.0890]	0.0144	0.0295	1.74 (5, 302.05)	0.125
3.Perceived neighborhood assets	Global perceived neighborhood-assets score	0.0636	[0.0206, 0.1258]	0.0388	0.0269	8.40 (1, 299.70)	0.004

Models were hierarchical and cumulative; each model retained all predictors entered in the preceding model. R^2^ and adjusted R^2^ are estimates pooled across 50 imputed datasets. ΔR^2^ represents the descriptive difference between the pooled R^2^ point estimates of consecutive models. Statistical inference regarding the contribution of each added block was based on pooled D_1_ tests comparing nested models fitted to the multiply imputed datasets. CI, confidence interval; df, degrees of freedom. PA, peer attachment; PsyAd, psychosocial adaptation; EA, Emotional Attention; EC, Emotional Clarity; ER, Emotional Repair; Adj_R^2^, adjusted R^2^.

### 3.5 Sensitivity analysis

The sensitivity analysis indicated that, with 313 participants, eight predictors, a two-sided α level of 0.05, and 80% power, the final regression model could detect an incremental effect of approximately *f*^2^ = 0.0258, corresponding to an approximate ΔR^2^ of 0.0242. The observed contribution of perceived neighborhood assets was ΔR^2^ = 0.0269, equivalent to *f*^2^ ≈ 0.0287. The study was therefore adequately sensitive to an incremental effect of the observed magnitude, although smaller associations may not have been detected reliably.

Because multiple imputation included the dependent variable, the hierarchical models were re-estimated while restricting the analyses to the 301 participants whose life-satisfaction scores were originally observed. The association between perceived neighborhood assets and life satisfaction remained virtually unchanged, *B* = 0.0453, *SE* = 0.0157, 95% CI [0.0144, 0.0762], *p* = 0.0042. The corresponding descriptive increment was ΔR^2^ = 0.0268, and the pooled comparison remained statistically significant, D_1_ *F*(1, 290.02) = 8.35, *p* = 0.0042.

A second sensitivity analysis was conducted using the 289 participants with complete information on all primary model variables. Participants included in and excluded from this analysis did not differ significantly in age, Welch’s *t*(27.01) = 0.74, *p* = 0.463; sex, Fisher’s exact *p* = 1.000; or grade distribution, simulated Fisher’s exact *p* = 0.252.

In the complete-case analysis, perceived neighborhood assets remained positively associated with life satisfaction when HC3 heteroskedasticity-consistent standard errors were used, *B* = 0.0448, SE = 0.0165, 95% CI [0.0124, 0.0773], *p* = 0.0069. The estimate was closely aligned with the multiply imputed result. In the complete-case models, R^2^ increased from 0.0083 in the sociodemographic model to 0.0427 after adding the relational, adaptive, and emotional variables and to 0.0698 after adding perceived neighborhood assets. The corresponding adjusted R^2^ for the final model was 0.0433, and the descriptive contribution of perceived neighborhood assets was ΔR^2^ = 0.0271. The substantive conclusion was therefore unchanged across the imputed-data, observed-outcome, and complete-case analyses.

### 3.6 Regression diagnostics

Variance inflation factors in the complete-case final model ranged from 1.01 to 2.64, providing no indication of problematic multicollinearity. The Breusch–Pagan test did not indicate heteroscedasticity, BP = 6.89, *df* = 8, *p* = 0.548. Thirteen observations exceeded the Cook’s-distance screening threshold of 4/*n*, but the maximum Cook’s distance was 0.034. These observations were retained because the threshold was used to identify cases for examination rather than as an automatic exclusion criterion.

Visual inspection of the partial-association plot supported an approximately linear positive association between perceived neighborhood assets and life satisfaction. The LOESS smoother broadly followed the linear fit across most of the observed range, with modest divergence at the upper end of the neighborhood-assets distribution. Observations exceeding the Cook’s-distance screening threshold were distributed across the plot and did not appear to account for the overall positive pattern (Figure 1).

**FIGURE 1:**
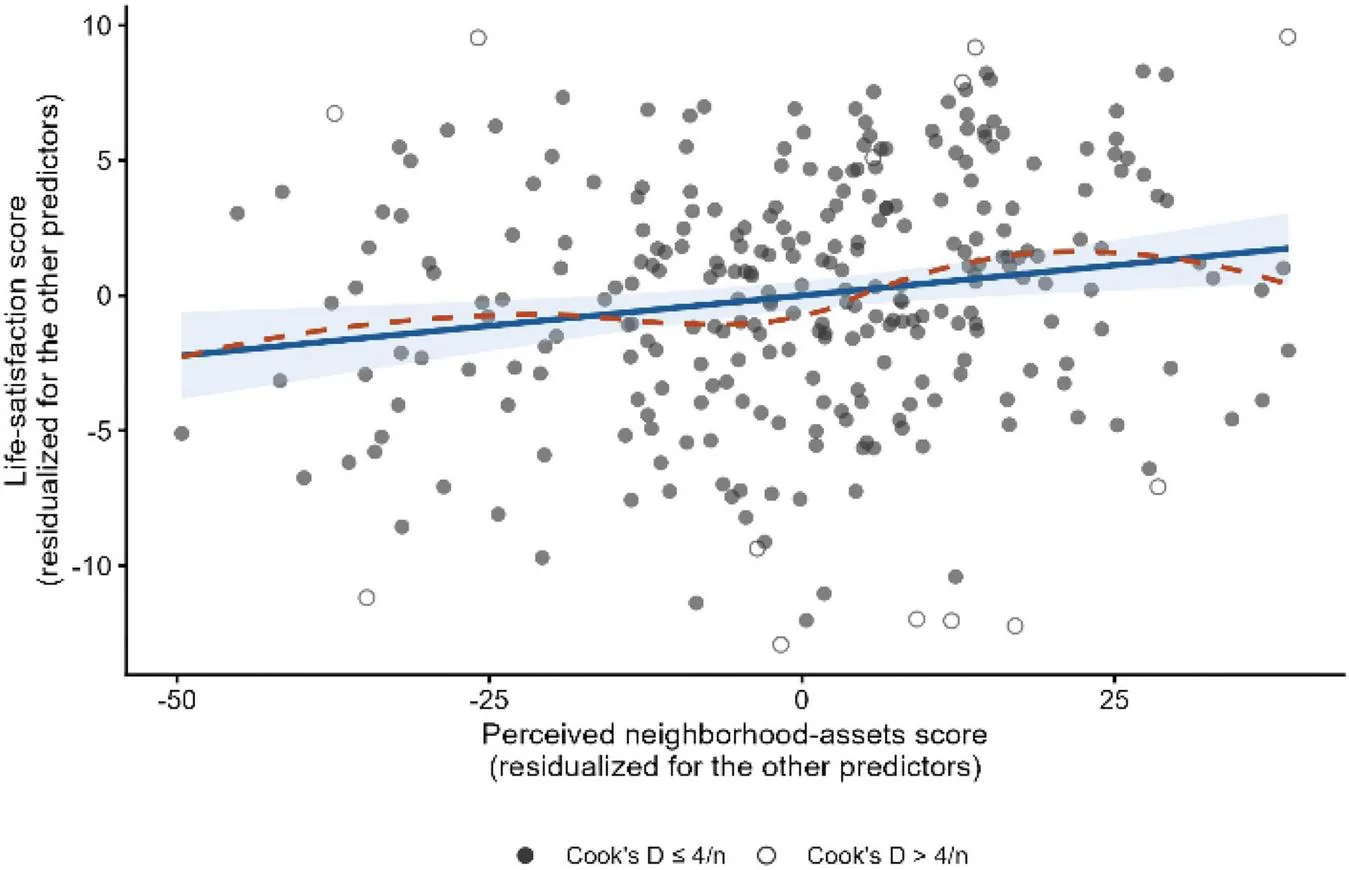
Partial association between perceived neighborhood assets and life satisfaction. The plot was constructed using the 289 participants with complete data for all variables in the final regression model. The vertical axis represents life-satisfaction scores residualized for age, sex, peer attachment, psychosocial adaptation, Emotional Attention, Emotional Clarity, and Emotional Repair; the horizontal axis represents neighborhood-assets scores residualized for the same covariates. The solid blue line represents the ordinary least-squares fit with its 95% confidence band, whereas the dashed orange line represents a locally estimated scatterplot smoother (LOESS). Open circles identify observations with Cook’s distance greater than 4/n. The figure provides a visual assessment of functional form and potentially influential observations; the primary statistical estimates were obtained from the 50 multiply imputed datasets.

### 3.7 Exploratory analysis of neighborhood-asset components

A *post hoc* exploratory analysis replaced the global neighborhood-assets score with its five component scores: youth support and empowerment, neighborhood attachment, perceived safety, informal social control, and activities for young people. The five components jointly accounted for additional variance in life satisfaction beyond age, sex, peer attachment, psychosocial adaptation, and the emotional dimensions, pooled D_1_ *F*(5, 296.09) = 2.45, *p* = 0.034. Pooled R^2^ increased from 0.0360 in the comparison model to 0.0775 in the component-level model, representing a descriptive ΔR^2^ of 0.0415.

At the bivariate level, life satisfaction was positively correlated with youth support and empowerment, ρ = 0.156, unadjusted *p* = 0.0066, adjusted *p* = 0.0219, and perceived safety, ρ = 0.153, unadjusted *p* = 0.0087, adjusted *p* = 0.0219. The correlations with neighborhood attachment, ρ = 0.105, adjusted *p* = 0.088; informal social control, ρ = 0.079, adjusted *p* = 0.183; and activities for young people, ρ = 0.116, adjusted *p* = 0.077, did not remain statistically significant after correction.

In the component-level regression model, perceived safety showed a positive coefficient before adjustment for multiple component-level comparisons, *B* = 0.1215, SE = 0.0554, β = 0.128, 95% CI [0.0124, 0.2306], unadjusted *p* = 0.029. Youth support and empowerment also showed a positive but statistically inconclusive coefficient, *B* = 0.0935, SE = 0.0499, β = 0.142, 95% CI [−0.0047, 0.1918], unadjusted *p* = 0.062. Neighborhood attachment, informal social control, and activities for young people were not independently associated with life satisfaction.

None of the five component-specific regression coefficients remained statistically significant after Benjamini–Hochberg correction: the adjusted *p*-values were 0.146 for perceived safety, 0.155 for youth support and empowerment, 0.692 for neighborhood attachment and informal social control, and 0.734 for activities for young people. These findings were therefore treated as exploratory rather than as evidence that a particular neighborhood component independently accounted for the association observed for the global score. Interpretation was further constrained by the limited internal consistency of the informal social control and activities-for-young-people components. Component-level descriptive statistics, reliability estimates, bivariate correlations, and regression coefficients are reported in Supplementary Tables 1A, B.

## 4 Discussion

This study examined whether adolescents’ appraisal of neighborhood assets was associated with life satisfaction after accounting for age, sex, peer attachment, psychosocial adaptation, and perceived emotional abilities. Perceived neighborhood assets made a statistically significant incremental contribution and were the only study variable independently associated with life satisfaction in the fully adjusted model. Adolescents who evaluated the assets available in their residential community more favorably also reported greater life satisfaction, although the magnitude of this association was modest. The final model explained 6.36% of the variance in life satisfaction, and perceived neighborhood assets accounted for a descriptive increment of 2.69% after the other variables had been entered. The standardized coefficient was similarly modest (β = 0.168): a one-standard-deviation difference in perceived neighborhood assets corresponded to an estimated difference of approximately 0.80 points on the 5–25 life-satisfaction scale. The association was closely reproduced when the analysis was restricted to participants whose life-satisfaction scores were originally observed and in the complete-case model using HC3 standard errors.

The findings are consistent with an ecological understanding of adolescent wellbeing in which young people’s evaluations of their lives are related to the social and physical environments in which their daily activities and relationships unfold. Previous research has linked perceived neighborhood resources, safety, belonging, collective efficacy, and opportunities for participation with adolescent adjustment and subjective wellbeing (Delgado et al., 2012; Varela et al., 2020; Steenwegen et al., 2026). The results also agree with evidence that subjective neighborhood assessments can capture aspects of place that are not represented fully by administrative or aggregate socioeconomic indicators, including whether local spaces are experienced as safe, accessible, socially supportive, and responsive to young people’s needs (Weden et al., 2008; Yoon, 2021).

The size of the association warrants measured interpretation. Most of the individual variation in life satisfaction remained unexplained, and perceived neighborhood assets should not be regarded as a dominant determinant of adolescent wellbeing. Multilevel studies have similarly found that neighborhood characteristics account for a small but detectable proportion of variation in adolescent life satisfaction or mental health after individual and family characteristics are considered (Marquez et al., 2024). The present study differs from such work because it did not estimate contextual differences between neighborhoods. It examined whether adolescents within a predominantly single-community school-based sample differed in life satisfaction according to their individual appraisals of their residential environments. The coefficient therefore represents an individual-level, within-sample association and should not be interpreted as an estimate of a neighborhood-level effect.

A modest individual-level association does not make neighborhood conditions inconsequential. Environmental conditions are experienced by many residents, and relatively small average differences may acquire practical relevance when exposure is widespread or sustained. The relationship may also vary with developmental stage, family circumstances, duration of residence, mobility, and the extent to which adolescents use community spaces. These possibilities were not examined in the present study.

The global neighborhood measure integrated youth support and empowerment, neighborhood attachment, perceived safety, informal social control, and activities for young people. Its association with life satisfaction may therefore reflect an overall experience of the residential environment rather than the contribution of one isolated characteristic. Safe and accessible spaces may facilitate contact with peers and participation in community activities; opportunities for participation may support belonging and agency; and confidence in community members may strengthen the perceived availability of support. These are plausible pathways connecting structural conditions and adolescents’ evaluations of their lives, but they were not tested as mediating mechanisms.

The exploratory component-level analysis provides only tentative differentiation within this global association. The five components jointly accounted for additional variance in life satisfaction. Youth support and empowerment and perceived safety showed small positive bivariate correlations with life satisfaction after correction for multiple comparisons, and perceived safety had a nominally significant coefficient in the adjusted component-level model. However, none of the five component-specific regression coefficients remained significant after Benjamini–Hochberg correction. The internal consistency of informal social control and activities for young people was also limited. The component-level results therefore do not establish that perceived safety—or any other single component—independently explains the association observed for the global score. They identify perceived safety and youth support and empowerment as priorities for replication with more reliable component measures and samples drawn from multiple communities.

Peer attachment, psychosocial adaptation, and the three emotional intelligence dimensions did not make statistically significant unique contributions to life satisfaction in the final model. These findings describe conditional associations within the specified regression and should not be interpreted as evidence that relational, adaptive, or emotional resources are unimportant. Peer attachment and Emotional Attention showed small bivariate correlations with life satisfaction, but neither remained statistically significant after correction for multiple comparisons. The emotional dimensions were moderately to strongly intercorrelated, although the variance inflation factors did not indicate problematic multicollinearity. Their overlapping variance may nevertheless have reduced the magnitude of their unique coefficients. The sensitivity analysis further indicated that the sample was adequate for detecting the incremental effect observed for perceived neighborhood assets but may not have detected smaller unique associations reliably. Differences between global and domain-specific measures, as well as unmeasured family and school resources, may also have contributed to these findings.

The territorial setting remains important for interpreting the neighborhood association. Participants attended a public school in a predominantly Afro-Colombian rural island community with documented limitations in sanitation, public services, transportation, healthcare access, and infrastructure. These characteristics describe the broader setting; they were not measured at the household level and cannot be used to classify the socioeconomic circumstances of individual participants or to attribute the observed association to ethnic identity. The results nevertheless show that variation in adolescents’ appraisal of the resources and opportunities available in their residential environments was related to life satisfaction within a territory facing longstanding structural constraints.

The four communities on Tierra Bomba Island share important sociohistorical characteristics and persistent limitations in public services, transportation, healthcare, sanitation, and infrastructure, but they should not be treated as interchangeable settings. Their proximity to mainland Cartagena, exposure to tourism, economic activities, heritage resources, transportation dynamics, and institutional presence differ. Such differences may shape adolescents’ opportunities to use community spaces and the meanings they assign to safety, attachment, participation, and available activities. The present findings may therefore be contextually relevant to other communities on the island, but they cannot establish that the same association or level of neighborhood appraisal would be observed in those settings.

The relevant psychological community may also extend beyond the adolescent’s immediate place of residence. Research on multiple psychological senses of community recognizes that individuals can experience belonging in relation to several nested or overlapping communities rather than a single territorial unit (Brodsky and Marx, 2001). In the present setting, these referents could include the local settlement, Tierra Bomba Island, or Cartagena. The questionnaire instructed participants to consider the residential community in which they lived, but the study did not assess which territorial level they considered most personally meaningful. It also did not record residence, length of residence, daily mobility, or the time spent using local spaces. A small, unquantified number of students were understood to commute from a neighboring island community. These factors may have influenced the relevance and interpretation of the residential environment for individual participants.

The cross-sectional design leaves the direction of the association unresolved. A favorable appraisal of neighborhood assets may support life satisfaction, adolescents with greater life satisfaction may evaluate their surroundings more positively, or both may reflect unmeasured family, school, and community conditions. Longitudinal and multisite studies are needed to distinguish these alternatives and to determine whether changes in objective neighborhood conditions are followed by changes in perceived neighborhood assets and subsequent life satisfaction.

### 4.1 Implications for policy and practice

The findings support including adolescents’ experiences of their residential environments in policies intended to promote wellbeing, while the cross-sectional design precludes concluding that neighborhood investment will necessarily increase life satisfaction. Government action in rural and island communities can address the material conditions that make supportive everyday environments possible: reliable water and sanitation, safe transportation, accessible healthcare and education, maintained public spaces, adequate lighting, environmental protection, recreational facilities, and sustained opportunities for cultural, sporting, and community participation. Infrastructure can also be accompanied by mechanisms through which adolescents participate meaningfully in decisions about the spaces and programs intended for them.

Evidence from neighborhood interventions provides qualified support for this approach. In Bogotá, the TransMiCable natural experiment evaluated an integrated intervention combining improved transportation with investments in public space and neighborhood infrastructure in a low-income settlement. The intervention was followed by improvements in quality of life and general health among women living in the intervention area, although comparable differential improvements were not observed among men (Baldovino-Chiquillo et al., 2025). This study indicates that changes in mobility and the built environment can be accompanied by improvements in perceived quality of life, while its sex-specific findings caution against assuming uniform benefits across population groups.

A synthesis of 54 studies evaluating 29 neighborhood policies found that place-based interventions—including housing refurbishment, greening, recreational infrastructure, and broader neighborhood regeneration—were more likely to produce beneficial short-term mental-health and wellbeing outcomes than policies based primarily on residential relocation (Wong et al., 2026). Effects varied by age, sex, intervention type, and follow-up period, and the evidence based specifically on validated measures of positive wellbeing was limited. Improvements in life satisfaction were not consistent across all interventions. Structural investment should therefore be understood as a potentially enabling condition whose psychological benefits may depend on implementation, accessibility, maintenance, social inclusion, and alignment with residents’ priorities.

Physical provision alone may have limited value when young people experience spaces as unsafe, inaccessible, poorly maintained, or disconnected from their interests. Policies can combine infrastructure improvements with community-based programming, youth participation, environmental stewardship, and channels through which adolescents can communicate their priorities to local institutions. In an island setting, such an approach also requires attention to geographic connectivity and continuity of services. Participation should extend beyond consultation and provide young people with identifiable roles in the design, monitoring, and evaluation of community initiatives.

These implications extend beyond the health sector. Adolescent life satisfaction may be addressed through coordinated decisions in education, transportation, sanitation, housing, environmental management, recreation, and public safety. Policy evaluations should include indicators reported directly by adolescents alongside objective measures of infrastructure and service access. Repeated assessments could determine whether changes in structural conditions are followed by changes in neighborhood appraisal and whether those changes precede improvements in wellbeing. Analyses according to sex, age, residence, mobility, and patterns of community-space use would also help identify unequal or unintended effects.

### 4.2 Strengths and limitations

This study contributes evidence from an adolescent population and a Colombian island community that has received limited attention in neighborhood and wellbeing research. The analysis considered demographic, relational, adaptive, emotional, and contextual variables simultaneously. Scale scoring was audited against the corresponding instrument instructions; internal consistency was evaluated using Cronbach’s alpha and polychoric total omega; remaining missing scores were addressed through multiple imputation; and the principal association was evaluated using observed-outcome and complete-case sensitivity analyses. The result was similar across these analyses and when HC3 standard errors were used. The component-level analysis was explicitly treated as exploratory and subjected to multiplicity correction.

Several limitations should be considered. First, the cross-sectional design precludes conclusions about temporal order and causality. Household socioeconomic circumstances, family structure, length of residence, actual use of community spaces, daily mobility, exposure to community violence, and access to family and school resources were not measured. These variables may influence both neighborhood appraisal and life satisfaction and therefore represent potential sources of residual confounding.

Second, participants were recruited by convenience from a single public school. The sample was drawn from nine intact classes, but classroom and school-schedule identifiers were not retained. Within-class dependence could therefore not be estimated, and cluster-adjusted standard errors could not be calculated. If residual outcomes were correlated within classrooms, the reported standard errors may be somewhat underestimated. The study also cannot separate individual differences in appraisal from contextual variation between residential communities because it did not use a multisite or multilevel design.

Most participants were understood to reside in the local community, but residence was not recorded and a small, unquantified number commuted from a neighboring island community. The sample should consequently be understood as school based rather than exclusively residential. It does not represent adolescents who were not attending school, whose residential experiences and life circumstances may differ. The four principal communities on Tierra Bomba Island share structural and sociohistorical conditions, but they also differ in connectivity, tourism, economic activities, infrastructure, and institutional presence. The findings may be contextually informative for the island, but statistical generalization to its other communities is not warranted. Replication across Bocachica, Punta Arena, Tierra Bomba, and Caño del Oro, together with comparisons involving socioeconomically disadvantaged communities in mainland Cartagena, would clarify which patterns are shared and which are specific to particular island settings.

Third, all constructs were reported by the same participants during the same administration sessions. Shared method variance and respondents’ general evaluative tendencies may therefore have increased some observed associations. Administration took place collectively in classrooms, although students completed the questionnaires individually and trained personnel were available to answer questions. This procedure may still have limited the detection of individual comprehension difficulties or inattentive responding. Response-time information was unavailable, and no screening based on invariant or repetitive response patterns was conducted. The final TMMS-24 item appeared separately from the preceding items because of the questionnaire layout, which should be considered when interpreting the comparatively greater item-level missingness in Emotional Repair. The reasons for individual omissions were not recorded.

Fourth, the instruments had been used previously in Spanish-speaking adolescent or Colombian samples, but no cognitive interviews, formal cross-cultural adaptation procedure, or measurement-invariance analysis was conducted specifically for this predominantly Afro-Colombian rural island population. Score comparability with the instruments’ original validation samples was therefore assumed rather than demonstrated. The SWLS used five response categories instead of the original seven, and its total scores are not directly comparable with scores obtained using the original response format.

The SWLS showed acceptable internal consistency according to Cronbach’s alpha and adequate consistency according to polychoric total omega. Its reliability was nevertheless somewhat lower than that of several predictor measures. Measurement error in the outcome may have reduced statistical precision and the proportion of variance explained, which should be considered when interpreting the modest model R^2^. The limited internal consistency of the informal social control and activities-for-young-people components also restricts interpretation of their exploratory coefficients.

Fifth, sex was self-reported using only the categories male and female, and gender identity was not assessed. Gender-diverse students could therefore not be represented. Finally, multiple imputation assumed that the remaining missing scores were missing at random conditional on the variables included in the imputation model. This assumption cannot be verified empirically. The consistency of the principal estimate in the observed-outcome and complete-case analyses reduces concern that the treatment of missing scores determined the finding, but it does not eliminate the possibility of bias from nonrandom missingness.

## 5 Conclusion

A more favorable appraisal of neighborhood assets was associated with greater life satisfaction among adolescents attending a public school in a rural island community of Cartagena. The association remained after demographic, relational, adaptive, and emotional characteristics were considered and was reproduced across sensitivity analyses, although its magnitude and the overall explanatory power of the model were modest. The findings concern individual differences in neighborhood appraisal within a predominantly single-community school-based sample and do not establish a causal or between-community contextual effect.

The results place adolescents’ experience of their residential environments within the study of subjective wellbeing and support further investigation of policies that combine reliable infrastructure, safe and accessible public spaces, community resources, and meaningful youth participation. Establishing whether such changes improve life satisfaction will require longitudinal, multisite, and intervention-based research that measures both structural conditions and young people’s perceptions of them.

## Data Availability

The raw data supporting the conclusions of this article will be made available by the authors, without undue reservation.

## Supplementary material

Supplementary Figure 1 Diagnostic plots for the multiple-imputation procedure. **(A)** Trace plots of the mean imputed values across 20 iterations for each of the 50 imputed datasets. The absence of persistent trends or systematic separation among the chains supports adequate convergence. **(B)** Density distributions of the observed (blue) and imputed (orange) scale scores. The distributions were broadly comparable, and all imputed values fell within the corresponding observed ranges. EmoAt, Emotional Attention; EmoRe, Emotional Repair; NAS, perceived neighborhood assets; PAS, peer attachment; SWLS, Satisfaction With Life Scale.
