# Supplementary material for: Perceived neighborhood assets and life satisfaction among adolescents attending school in a rural insular community
Source: Front Psychol. 2026 Oct 14;17:1992746. doi: 10.3389/fpsyg.2026.1992746 (PMC13713363; doi:10.3389/fpsyg.2026.1992746)
Supplement: Supplementary file 1 [file Table_1.docx]

**Supplementary Table S1.** Exploratory analyses of the neighborhood-asset components

**Panel A. Descriptive statistics and internal consistency**

| Component | Items | Score n | M | SD | Median | Observed range | Theoretical range | Reliability n | α | Polychoric ωₜ |
| --- | --- | --- | --- | --- | --- | --- | --- | --- | --- | --- |
| Youth support and empowerment | 6 | 313 | 26.03 | 7.28 | 27 | 6–42 | 6–42 | 305 | .787 | .809 |
| Neighborhood attachment | 4 | 306 | 19.24 | 5.63 | 20 | 4–28 | 4–28 | 306 | .809 | .841 |
| Perceived safety | 4 | 304 | 10.37 | 5.03 | 10 | 4–28 | 4–28 | 304 | .702 | .784 |
| Informal social control | 4 | 301 | 21.26 | 4.33 | 22 | 4–28 | 4–28 | 301 | .582 | .672 |
| Activities for young people | 4 | 306 | 17.26 | 4.91 | 17 | 4–28 | 4–28 | 306 | .483 | .523 |

*Note.* Score n denotes the number of participants with an available component score. Reliability n denotes the number with complete item-level responses used to estimate α and polychoric ωₜ.

**Panel B. Bivariate correlations and component-level regression coefficients**

| Component | Bivariate n | ρ with SWLS | Raw p | BH-adjusted p | B | SE | β | 95% CI for B | Model raw p | Model BH-adjusted p |
| --- | --- | --- | --- | --- | --- | --- | --- | --- | --- | --- |
| Youth support and empowerment | 301 | .156 | .0066 | .0219 | .0935 | .0499 | .142 | [−.0047, .1918] | .0620 | .1550 |
| Neighborhood attachment | 296 | .105 | .0707 | .0884 | −.0348 | .0586 | −.041 | [−.1500, .0805] | .5534 | .6918 |
| Perceived safety | 294 | .153 | .0087 | .0219 | .1215 | .0554 | .128 | [.0124, .2306] | .0292 | .1460 |
| Informal social control | 289 | .079 | .1831 | .1831 | .0439 | .0734 | .040 | [−.1006, .1885] | .5498 | .6918 |
| Activities for young people | 295 | .116 | .0459 | .0766 | .0236 | .0694 | .024 | [−.1130, .1602] | .7340 | .7340 |

*Note.* Bivariate p-values were adjusted across the five component–SWLS correlations. Model p-values were adjusted separately across the five component-specific regression coefficients. The component-level model adjusted for age, sex, peer attachment, psychosocial adaptation, Emotional Attention, Emotional Clarity, and Emotional Repair. The five components jointly improved the model, pooled D₁ F(5, 296.09) = 2.45, p = .034; pooled R² increased from .0360 to .0775 (descriptive ΔR² = .0415). BH = Benjamini–Hochberg; SWLS = Satisfaction With Life Scale.
